# Methane Conversion Under Mild Conditions Using Semiconductors and Metal-Semiconductors as Heterogeneous Photocatalysts: State of the Art and Challenges

**DOI:** 10.3389/fchem.2021.685073

**Published:** 2021-06-30

**Authors:** Eliane Ribeiro Januario, Patrícia Ferreira Silvaino, Arthur Pignataro Machado, Jorge Moreira Vaz, Estevam Vitorio Spinace

**Affiliations:** Instituto de Pesquisas Energéticas e Nucleares – IPEN-CNEN/SP, Centro de Células a Combustível e Hidrogênio, Cidade Universitária, São Paulo, Brazil

**Keywords:** methane, photocatalysts, heterogeneous, semiconductor, metal-semiconductor

## Abstract

The processes currently used in the chemical industry for methane conversion into fuels and chemicals operate under extreme conditions like high temperatures and pressures. In this sense, the search for methane conversion under mild conditions remains a great challenge. This review aims to summarize the use semiconductors and metal-semiconductors as heterogeneous photocatalysts for methane conversion under mild conditions into valuable products. First, a brief presentation of photochemical conversion of methane is provided and then the focus of this review on the use of heterogeneous photocatalysts for methane conversion are described. Finally, the main challenges and opportunities are discussed.

## Introduction

Methane is the principal component of natural gas having the highest C/H ratio among the hydrocarbons and is an important source of carbon (C_2+_), carbon monoxide and hydrogen for the chemical industry (Horn and Schlögl, 2015; Olivos-Suarez et al., 2016). However, methane is one of the most stable molecule and methane conversions are thermodynamically unfavorable at 298°K due to the positive and large value of the Gibbs free energy of formation (ΔG > 0). Thus, high reaction temperatures are required to transform methane into more valuable and useful chemicals and to promote such reactions suitable catalysts are also necessary to reduce the activation energy (Yuliati and Yoshida, 2008). In this manner, further development of technology for methane conversion under mild conditions in order to reduce the energy consumption and the environmental impacts are highly desired (Horn and Schlögl, 2015; Olivos-Suarez et al., 2016).

## Thermocatalytic Conversion of Methane

The steam reforming of methane (SRM) is the main process currently used for hydrogen and carbon monoxide production (Eq. 1), which are key intermediates in the chemical industry. This reaction is endothermic and the catalytic process is carried in the temperature range of 970–1100°K and pressure up to 3.5 MPa (Horn and Schlögl, 2015; Olivos-Suarez et al., 2016).

$$\begin{array}{l} С{Н}_{4}+H_{2}\mathrm{Ο⇆СΟ}+3H_{2}\\ \Delta {Н}_{298K}=+206\;\mathrm{KJ}mol^{-1} \end{array}$$ (1)

The methane steam reforming reaction is an indirect route for transforming methane into higher value-added chemicals such as ammonia, methanol and liquid fuels.

A direct route of high industrial interest is the oxidative coupling of methane (OCM) to ethane and ethylene (Eqs 2, 3). This is a highly exothermic reaction and occurs in the presence of several metal oxide catalysts at temperatures between 773 and 1273°K (Olivos-Suarez et al., 2016; Gao et al., 2019).

2CH4+12O2→C2H6+H2ΟΔН1073K=−1465 KJmol−1 (2)

$$\begin{array}{l} 2CH_{4}+O_{2}\to C_{2}H_{4}+H_{2}Ο\\ \Delta {Н}_{1073K}=-1163\;\mathrm{KJ}mol^{-1} \end{array}$$ (3)

The main products of these reactions are ethane and ethene in addition to CO_2_ and CO, but due to difficult control of this process it was not industrially applied yet (Horn and Schlögl, 2015; Olivos-Suarez et al., 2016).

In the non-oxidative coupling of methane (NOMC), methane is directly converted into ethane and/or ethylene and hydrogen (Eqs 4, 5) in the presence of a catalyst (Zeng et al., 2018).

$$\begin{array}{l} CH_{4}⇆1/2C_{2}H_{6}+1/2H_{2}\\ \Delta {Н}_{298K}=+65\;\mathrm{KJ}mol^{-1} \end{array}$$ (4)

$$\begin{array}{l} \;CH_{4}⇆1/2C_{2}H_{4}+H_{2}\\ \Delta {Н}_{298K}=+101\;\mathrm{KJ}mol^{-1} \end{array}$$ (5)

However, the two-stage process taking place at different temperatures is difficult to operate and the thermodynamic limitations cause extremely low activity (Tang et al., 2014).

Another direct route of high industrial interest is the direct oxidation of methane to methanol (Eq. 6) and/or formaldehyde (Eq. 7) using heterogeneous catalysts (Song et al., 2019a).

CH4+12O2→CH3OHΔН298K=−126 KJmol−1 (6)

$$\begin{array}{l} CH_{4}+O_{2}\to CH_{2}O+H_{2}O\\ \Delta {Н}_{298K}=-368\;\mathrm{KJ}mol^{-1} \end{array}$$ (7)

In this case, although the reactions are thermodynamically favored, they are also difficult to control because the products formed are more reactive than methane leading to over-oxidation products, mainly CO_2_. Thus, despite decades of research partial oxidation reactions of methane and oxygen are yet difficult to control and both are even further away from practical applications (Horn and Schlögl, 2015; Olivos-Suarez et al., 2016).

## Photocatalytic Conversion of Methane

An alternative to minimize energy use and environmental impacts is to perform the methane conversions at milder temperatures and under conditions that are easier to control, which is possible using photoenergy instead of the thermal energy Photochemical reactions proceed at low temperatures because the photoenergy surpasses the activation energy of the chemical reactions (Yuliati and Yoshida, 2008). On the other hand, despite this photoenergy is too low to break directly the C-H bond (434 kJ mol^−1^) of methane, the use of photocatalysts may favor its activation at mild conditions in thermodynamically unfavorable reactions (ΔG > 0) (Yuliati and Yoshida, 2008). For thermal catalytic reactions, the forward and backward reactions continue to occur at equilibrium while for photocatalytic reactions they can be considered as two different reactions requiring different energies and occurring by different mechanisms. Thus, the photocatalytic reaction break the thermodynamic equilibrium by decreasing or eliminating the backward reaction (Yuliati and Yoshida, 2008).

In the photocatalytic reactions, semiconductors are used as photocatalyts and when the photoenergy is greater than the band gap (E_g_) of the semiconductor the photoexcitation occurs resulting in the promotion of an electron (*e* ^*−*^) from the valence band (VB) to the conduction band (CB) forming a hole (*h* ^*+*^) in the latter. The excited electrons and holes have potential enough to promote reduction and oxidation reactions (half reactions), respectively (Yuliati and Yoshida, 2008; Song et al., 2019b).

The photocatalysis involves a sequence of processes comprising the following steps: photon absorption, charge separation, charge carrier diffusion and transport, surface redox reactions, and reactant or product mass transfer. In order to obtain a good performance, all processes must take place efficiently (Takanabe, 2019). Thus, photocatalytic reaction is affected by the absorption of light, the separation and migration of electron–hole pairs and the chemical reaction on the catalyst surface and its efficiency can be improved by increasing the effective photon absorption, promoting charge separation and creating more active sites (Yuliati and Yoshida, 2008; Liu et al., 2017).

The photocatalytic non-oxidative coupling of methane at around room temperature was first described in 1998. Using TiO_2_–SiO_2_ and Ga_2_O_3_ as photocatalysts under UV irradiation it was possible to obtain ethane as the main product, however conversions were very low (Yuliati and Yoshida, 2008). The use of solar energy and a photocatalyst would also be an ideal method to produce hydrogen from water as an environmentally benign fuel (Shimura et al., 2010). However, this reaction has a positive value of Gibbs free energy (ΔG > 0) which makes it difficult to occur. To circumvent this barrier, the a sacrificial reagent is used to consume the photogenerated holes reducing the reverse reactions (Shimura et al., 2010). Sacrificial reagents normally used were some kinds of carbon-related materials like sugar, starch, cellulose, coal and some compounds such as methanol, ethanol, ethylene and carbon monoxide. The production of hydrogen using methane as a “sacrificial reagent” was described by Yoshida and collaborators (Yoshida et al., 2007) in a reaction interpreted as a photocatalytic steam reforming of methane (PSRM), where Pt-loaded on semiconductors (NaTaO_3_ and TiO_2_) were used as photocatalysts to convert methane and water.

## Heterogeneous Photocatalysts for Methane Conversion

Although activation of methane using heterogeneous photocatalysts are quite interesting from the academic and technological point of view few papers have been published on the subject in the last years (Wu et al., 2021).

### Semiconductor Photocatalysts

In this section the results of methane conversion using semiconductors as photocatalysts are presented (Table 1).

**TABLE 1:** Methane conversion using semiconductors as photocatalysts arranged in the order of publication year.

Entry	Reactor	Source	Photocatalyst	Photocatalyst amount	CH_4_ inlet	H_2_O	CH_4_ conv	t	T	Products formed	References
(1)	Closed quartz reaction vessel (82 ml)	Xe lamp 250 W	SiO_2_.Al_2_O_3_ spread on the flat bottom of the vessel	1.0 g	100 μmol (21 torr)	-	5.9%	18 h	310°K	C_2_H_6_ (3.5) C_2_H_4_ (0.4) C_3_H_8_ (0.85) C_3_H_6_ (0.3).μmol	Kato et al. (1998)
(2)	Closed quartz reactor (30 cm^3^)	300 W Xe lamp	Ga_2_O_3_	0.2 g	CH_4_ (200 µmol)	-	-	3 h	314°K	C_2_H_6_ = 0.51 µmol	Yuliati et al. (2008a), Yuliati et al. (2008b)
										C_2_H_4_ = 0.01 µmol	
										C_3_H_8_ = 0.05 µmol	
										H_2_ = 0.74 µmol	
(3)	Commercial photochemical reactor (Ace Glass)	Hg lamp	-β zeolite (BEA)	0.50 g L^−1^	CH_4_/He (20%) ∼22 ml min^−1^	300 ml	-	120 min	70°C	Bi-V-BEA	Murcia-López et al. (2017)
										CH_3_OH = 11 μmol g^−1^ h^−1^	
		450 W	-Bi-V- BEA							C_2_H_6_ = 2.5 μmol g^−1^ h^−**1**^	
										CO_2_ = 150 μmol g^−1^ h^−1^	
(4)	Home made fixed bed tubular quartz reactor 500 ml	Hg lamp	Ga_2_O_3_/AC (15 wt%)	0.20 g	CH_4_:O_2_:N_2_	-	91.5%	150 min	25°C	CO_2_	Wei et al. (2017)
		10 W			1.56 mmol L ^−1^ CH_4_						
(5)	Gas-tight glass cell	300-W Xe lamp	CeO_2_ treated 500–1,100°C	2 mg	0.2 bar CH_4_	15 ml	-	-	-	CH_3_CHO = 1.0 μmol g^−1^ h^−1^	Du et al. (2020)
										CH_3_CH_2_OH = 11.4 μmol g^−1^ h^−1^	
(6)	Autoclave of 130 ml equipped with a quartz window	300 W Xenon lamp	TiO_2_, Fe_2_O_3_, NiO, CuO, ZnO, WO_3_	20 mg	3 MPa CH_4_ and Fe^2+^/H_2_O_2_ (Fenton)	20 ml	0.39%	1 h	30°C	Fenton/TiO_2_	Zeng et al. (2020)
										CH_3_OH = 471 μmol g^−1^ h^−1^	
										HCOOH = 34 μmol g^−1^ h^−1^	
										CH_3_COH = 53 μmol g^−1^ h^−1^	

The non-oxidative coupling of methane proceeding under photoirradiation at around room temperature in the presence of silica-alumina as photocatalyst was first described in 1998 (Table 1, entry 1). It was detected C_2_–C_4_ alkanes in the gas phase and smaller amounts of C_2_–C_6_ alkanes and alkenes were detected after desorbing from the catalyst upon heating. It is important to highlight that CO and CO_2_ were not detected and that in the absence of irradiation and/or catalysts, no products were observed. A 5.9% methane conversion was obtained after 18 h of irradiation resulting in an activity of 0.327 μmol h^−1^ (Kato et al., 1998).

Yoshida and collaborators using gallium oxide (Ga_2_O_3_) converted photocatalytically methane mainly in ethane and hydrogen at 314°K (Table 1, entry 2). It was observed that the conversion exceeded the equilibrium at this temperature indicating that Ga_2_O_3_ photocatalysts can selectively promote the forward reaction of the non-oxidative coupling of methane which is an advantage of the photocatalytic reaction (Yuliati et al., 2008a; Yuliati et al., 2008b).

Beta zeolite (H-BEA) was active for the oxidation of methane under UVC light) forming almost exclusively to CO_2_ as the total oxidation product (Table 1, entry 3. The addition of V and Bi-V to the zeolite framework decreased the amount of acid sites due to the formation of V_2_O_5_ on the surface and improved the selectivity to less oxidized products. The addition of low Bi amount favored the formation of a BiVO_4_/V_2_O_5_ heterojunction that acts as a visible light photocatalyst decreasing significantly the formation of CO_2_ when compared to H-BEA and leading almost exclusively the formation of methanol instead of the ethylene formation (Murcia-López et al., 2017).

The photocatalytic decomposition of methane to CO_2_ under UV irradiation was studied using β-Ga_2_O_3_ supported on activated carbon (Table 1, entry 4). The photocatalytic activity was dependent on weight ratio of Ga_2_O_3_ being 15 wt% exhibited the best activity, which was more than sixfold of TiO_2_ P25 benchmark photocatalyst. This increase of activity was ascribed to a strong adsorption capacity and improved separations of photoelectrons (*e* ^*−*^) and vacancies (*h* ^*+*^) pairs. The authors consider this method promising to remove low concentrations of methane (Wei et al., 2017).

Ceria nanoparticles with abundant oxygen vacancies were prepared at high temperatures under argon atmosphere, showing the highest oxygen vacancy concentration at 1,100°C (Table 1, entry 5). This material showed high activity for photocatalytic methane conversion leading to the formation of ethanol and acetaldehyde, being ethanol formed with 91.5% of selectivity. The aldehyde formation was attributed to the subsequent oxidation of the formed ethanol (Du et al., 2020).

The photocatalytic conversion of methane to oxygenated compounds at room temperature was studied combining the Fenton chemistry (Fe^2+^/H_2_O_2_) and different semiconductors like TiO_2_, Fe_2_O_3_, NiO, CuO, ZnO and WO_3_ (Table 1, entry 6). For comparative purposes, the Fenton reaction (FR), the traditional photocatalytic process (PCR) and the junction photo-Fenton reaction (PCFR) using TiO_2_ as photocatalyst were performed. PCFR process showed the best results producing large amounts of methanol (up to 471 μmol g^−1^ h^−1^) with a selectivity of 83%, being the others products formic acid (HCOOH) and acetaldehyde (CH_3_CHO). The semiconductors NiO, CuO and WO_3_ also showed good methane conversions in the PCFR process; however producing HCOOH as the principal product with low selectivity values for methanol (in the range of 15–30%). Fe_2_O_3_ and ZnO showed low activities for methane conversion. The authors showed that the increase of PCFR process activity is a result of the synergism between Fenton reaction and photocatalysis involving active radicals (hydroxyl and superoxide) and charge carriers (excited electrons and holes) (Zeng et al., 2020).

### Metal-Semiconductor Photocatalysts

Various studies have been shown that the integration of metals with semiconductors results in the formation of photocatalysts with improved activities that has been attributed to several factors: (Reddy et al., 2019; Wu et al., 2021).

1) the activation of methane by metals,
2) by displaying plasmonic oscillations that results in localized surface plasmon resonance (LSPR) effect,
3) to the improvement of charge separation (photoelectrons and holes) pulping the photoelectrons to surface for reactions (Schottky junction).

In Table 2 it was shown the use metal-semiconductor as photocatalysts for methane conversion.

**TABLE 2:** Methane conversion using metal-semiconductors as photocatalysts arranged in the order of publication year.

Entry	Reactor	Source	Photocatalyst	Photocatalyst amount	CH_4_ inlet	H_2_O	CH_4_ conv	t	T	Products formed	References
(1)	Commercial 1 L quartz	High pressure Hg lamp 350 W	WO_3_ doped with Pt, La or Cu	-	CH_4_ (5 ml min^−1^) 1.0 MPa pressure experiment, methyl viologen (MV) as electron transfer	750 ml	∼4%	-	370°K	WO_3_ doped La	Taylor (2003)
										CH_3_OH = 1.7 g g^−1^ h^−1^	
(2)	Fixed-bed flow reactor - quartz cell (60 × 20 × 1 mm^3^)	Xe lamp 300 W	Doped and undoped Ga_2_O_3_ with Me (Pt,Rh, Au,Pd, Ni)	Catalyst (0.8 g) quartz granules (0.7 g)	H_2_O_vapour_ (30 μmol min^−1^)/CH_4_ (120 μmol min^−1^) with Ar carrier (50 ml min^−1^)	Vapor	-	-	308°K	Pt/Ga_2_O_3_	Shimura et al. (2010)
										H_2_ = 0.55 μmol min^−1^	
(3)	airtight	High pressure Hg lamp150 W	Metal exchanged ETS-10	0.2 g spread on the wall of the reactor	200 μmol CH4	-	14.9%	5 h	RT	Ga^3+^ ETS-10	Li et al. (2012)
	quartz reactor (20 cm^3^)									C_2_H_6_ ∼20 mmol h^−1^ g^−1^	
(4)	Self-fabricated photocatalytic reactor –batch process	Laser energy 100 MJ, 355 nm	5 wt% Ag_2_O/WO_3_	0.30 g	100 ml/min for a period of 15 min after expelling oxygen by argon purging	70 ml	-	1.5 h	-	CH_3_OH 13 μmol min^−1^	Hameed et al. (2014)
										H_2_ ∼ 42 μmol min^−1^	
(5)	Quartz photochemical reactor (Ace Glass) of 500 ml	Medium-pressure mercury lamp (Ace Glass)–UV C	La doped mesoporous WO_3_	0.30 g	A mixture of methane (4.5 ml min^−1^) and helium (17.9 ml min^−1^) sparged continuosly	300 ml	-	2 h	55°C	CH_3_OH 9 μmol min^−1^	Villa et al. (2016)
										C_2_H_6_ 1 μmol min^−1^	
										CO_2_ 10 μmol min^−1^	
(6)	Homemade fixed-bed pyrex reactor of 450 ml	Xe lamp 300 W. 200 mWcm^−2^	Ag/ZnO (0.1 wt%)	0.50 g	95% N_2_	-	0.35%	-	RT	C_2_H_6_ (89.5% Selectivity). C_2_H_4_ (10.5% Selectivity)	Chen et al. (2016)
					5% CH_4_						
					10 ml min^−1^ (free O_2_)						
(7)	gas-liquid-solid system	UV lamp	Pt/TiO_2_	0.075 g	CH_4_ 10 ml min^−1^	75 ml	1.6%	6 h	25°C	C_2_H_6_ = 25 μmol	Yu et al. (2017)
		254 nm	1.5 wt% Pt							CO_2_ = 35 μmol. H_2_ = 180 μmol	
(8)	Gas-liquid-solid system	UV lamp	Pd/TiO_2_ (1.5 wt%)	0.075 g	CH_4_ 10 ml min^−1^	75 ml	1.0%	6 h	25°C	C_2_H_6_ = 25 μmol	Yu and Li (2017)
		254 nm								CO_2_ = 10 μmol. H_2_ = 55 μmol	
(9)	Flow-type quartz reactor	Hg lamp	-TNT- Rh/TNT-Au/TNT-Au/TiO_2_	0.50 g	0.9 ml min^−1^ CH_4_, 28.2 ml min-1 Ar (30 ml min^−1^), 0.9 ml min^−1^ water vapor stream	Vapor	-	-	403°K	Rh/TNT_2_	László et al. (2018)
		500 W								H_2_ =296 μmol g^-1^ h^-1^	
										C_2_H_6_ = 3.0 μmol g^-1^ h^-1^	
										CO_2_ = 41 μmol g^-1^ h^-1^	
(10)	Custom-made batch reactor 170 ml cell	300 W xenon lamp	Anatase TiO_2._ 0.12% Au/TiO_2_ 0.53% PdOx/TiO_2._ 0.69% PtO/TiO_2_. 0.29% Cu_2_O/TiO_2_ 0.33% FeO_x_/TiO_2_	0.010 g	CH_4_: Argon (1:19). 2 mM H2O2 solution (4 ml)	6 ml	14.9%	3 h	25°C	FeO_x_/TiO_2_	Xie et al. (2018)
										CH_3_OH =	
										1,056 µmol gcat^−1^	
(11)	Homemade stainless-steel batch reactor 230 ml	300 W Xe lamp equipped with a reflector	Pt, Pd, Au, Ag 0.1% supported on ZnO and TiO_2_	0.010 g	0.1 MPa O_2_	100 ml	-	4 h	25°C	Au/ZnO	Song et al. (2019a)
										CH_3_OOH = 123.4 μmol	
										CH_3_OH = 41.2 μmol	
					2 MPa CH_4_					HCHO = 86.3 μmol	
										CO = 0.4 μmol	
										CO_2_ = 11.6 μmol	
(12)	100 ml micro autoclave	Xe lamp 84.2 mW/cm^2^	0.3-1.5% Ag/TiO_2_	100 mg	CO_2_/CH_4_/Ar = 7.5/7.5/85 in terms of mole fraction)	-	-	2 h	-	C_2_H_4_ = 686 μmol g^−1^ h^−1^	Li et al. (2019)
					- pressure 2 MPa					CO = 1,149 μmol g^−1^ h^−1^	
(13)	Homemade stainless-steel batch reactor 250 ml	Xe lamp	ZnHPV TiO_2_	0.10 g	CH_4_ 0.3 MPa	-	-	6 h	RT	CO = 429 μmol g^−1^ h^−1^	Yu et al. (2019)
		400 W			Air 0.1 MPa					CO_2_ = 85 μmol g^−1^ h^−1^	
(14)	Photochemical reactor	Xe lamp 500 W	Cu -PCN	20 mg	CH_4_ 10 ml min^−1^ and N_2_ 90 ml min^−1^	25 ml	-	1 h	RT	C_2_H_5_OH = 21 μmol g^−1^ h^−1^	Zhou et al. (2019)
										CH_3_OH = 5.5 μmol g^−1^ h^−1^	
										C_2_H_6_ = 13.9 μmol g^−1^ h^−1^	
										H_2_ = 7.0 μmol g^−1^ h^−1^	
										CO = 2.7 μmol g^−1^ h^−1^	
(15)	Stainless-steel equipped with a quartz window (flow system)	365 nm light by a 40 W LED	Cu_X_/PtTiO_2_	100 mg	O_2_: CH_4_ = 1: 400	50 ml	-	-	RT	C_2_H_6_ + C_2_H_4_ (6.8 μmol h^−1^)	Li et al. (2020)
					10% CH_4_						
(16)	Homemade reactor	300 W Xe lamp with an AM 1.5G filter (100 mW cm^−2^)	M/TiO_2_ (M = Au,Pt,Ir	5 mg deposited onto diffuse reflecting holder	10% CH_4_ in argon	-	-	-	RT	Au/TiO_2_	Lang et al. (2020)
			Ag,Pd,Rh,Ru)							C_2_H_6_ = 81.7 µmol g_cat_^−1^ h^−1^	
(17)	Harrick reactor	White light illumination 19.2 W cm^−2^	Cu–Ru/MgO-Al2O3	∼1.5 mg	CH_4_ 8 ml min^−1^	-	275 µmol CH_4_ g^−1^ s^−1^	2 h	RT	CO: H_2_ (1:1)	Zhou et al. (2020)
					CO_2_ 8 ml min^−1^						
(18)	33 ml custom-made tube reactor	300 W xenon lamp	Pd-modified Au/ZnO	2.0 mg dropped on conductive glass	0.5 ml of CH_4_	-	536 µmol CH_4_ g^−1^	8 h	RT	C_2_H_4_ = 102.3 μmol g^-1^	Jiang et al. (2021)

Taylor considered the use of abundant and relatively low-cost reactants like light, water and methane was an attractive process to produce methanol and hydrogen (Table 2, entry 1). Using tungsten oxide (WO_3_) doped with Pt, La or Cu and a mixture of CuLa as photocatalysts and an electron transfer reagent (methyl viologen dichloride) at 370°K and 1.0 MPa it was possible to convert methane and water to methanol and hydrogen as the main products and ethane, oxygen, formic acid and carbon dioxide formed by side reactions. The tungsten oxide doped with lanthanum photocatalyst exhibited the largest methane conversion (∼4%) and methanol yield of 1.7 g_cat_^−1^ h^−1^ (Taylor, 2003).

The photocatalytic steam reforming of methane (PSRM) was performed around room temperature using doped and undoped Ga_2_O_3_ loaded with metals (Pt, Rh, Au, Pd and Ni) (Table 2, entry 2). The Pt/Ga_2_O_3_ exhibited superior performance for PSRM reaction exhibiting higher hydrogen production. The order of the activity was Pt > Rh > Au > Pd > Ni and the activity of the Pt/Ga_2_O_3_ photocatalyst was further enhanced by the addition of metal ions into the bulk and/or on the surface of Ga_2_O_3_. (Shimura et al., 2010).

The photoactivation of the methane C–H bond was performed using metal ions-modified ETS-10. This material is a microporous ordered titanosilicate with a framework containing one-dimensional O–Ti–O–Ti–O semiconducting nanowires (diameter of 0.67 nm) (Table 2, entry 3). These structures are insulated from one another by the surrounding SiO_2_ matrix (Li et al., 2012). They considered that the combination of properties such quantum-confined titanate wires that improving the electron transfer, the uniform pore structure which allow the CH_4_ access, and the presence of extra framework cations (Na^+^, K^+^), renders ETS-10 attractive for photoactivation applications. Besides this, the incorporation of extra metal ions into ETS-10 through either ion exchange or isomorphous substitution enhances its photoactivity. Through ion exchange it was prepared a series of metal ions-ETS-10 (metal ions = Ga^3+^, Al^3+^, Zn^2+^, Fe^3+^ and Cu^2+^). The photocatalysts were tested at room temperature for methane conversion in the absence of water and oxygen and C_2_H_6_ was the principal product formed with smaller amounts of C_2_H_4_ and C_3_–C_4_ alkanes and alkenes. Ga^3+^-ETS-10 showed the best activity and after 5 h of reaction a conversion of 14.9% was observed corresponding to a methane conversion of around 29.8 mmol g_cat_^−1^ h^−1^. The authors associated the superior performance of Ga^3+^-ETS-10 to the presence of extraframework metal cations and the terminal Ti–OH groups on titanate wires, interacting with methane molecules under UV irradiation, leading to the cleavage of the methane C–H bond (Li et al., 2012).

The aqueous phase photocatalytic conversion of methane into methanol and hydrogen was investigated over Ag^+^-impregnated WO_3_ (Table 2, entry 4). The characterization by different techniques showed that Ag^+^ ions shared surface oxygen with WO_3_ forming Ag_2_O that improved the photons absorption. The presence of cations Ag^+^ at the surface of semiconductor WO_3_ enhanced the formation of •OH radicals and suppressed the charge carrier recombination process. On the other hand, the metal loading higher than 5% affect decrease the conversion process because photons are consumed preferentially in the dissociation of Ag_2_O. The photocatalytic process occured on the surface of the catalyst as well in the bulk that included water splitting and methanol formation. The measurement of evolved H_2_ and O_2_ in the gas phase and methanol in the liquid phase revealed that the produced methanol competes with water molecules for the photogenerated holes and undergoes further decomposition. The authors concluded that the yield of methanol could be enhanced by its removal after formation. (Hameed et al., 2014).

Based on the fact that ordered mesoporous metal oxides showing large surface area and good light harvesting can result in efficient catalysts for many photocatalytic applications, La-doped mesoporous WO_3_ was tested for the photocatalytic conversion of methane into methanol (Table 2, entry 5). La/WO_3_ was prepared in the following manner: a solution of LaNO_3_ was added to the ethanolic solution of phosphotungstic acid hydrate (precursor of WO_3_) and the resulting mixture was incorporated inside the host silica (KIT-6) that was further removed using HF. The photocalytic activities of mesoporous WO_3_ and La/WO_3_ were evaluated on the partial oxidation of methane to methanol with water under UVC-visible light irradiation at 55°C. The principal products formed were CH_3_OH, C_2_H_6_ and CO_2_ with minor quantities of H_2_, C_2_H_4_, ethanol and formaldehyde. The photocatalyst La/WO_3_ produced more methanol and less amounts of C_2_H_6_ and CO_2_ than pure WO_3_; although appreciable amounts of CO_2_ were produced for both catalysts. The superior performance of La/WO_3_ was mainly attributed to the formation of oxygen vacancies that increase H_2_O adsorption and modification of basic-acid properties resulting in higher formation of •OH radicals (Villa et al., 2016).

Nanoparticulate ZnO exhibited exceptional activity under ultraviolet and UV-Vis light illumination as photocatalyst for CH_4_ oxidation ((Table 2, entry 6). For comparison purposes, the performance of commercial ZnO with particle sizes (200–300 µm) and TiO_2_ P25 (benchmark photocatalyst), under the same conditions, exhibited mild and faint activity, respectively. Under visible light commercial ZnO and P25 showed no activity for CH_4_ oxidation. On the other hand, nanoparticulate ZnO still shown significant activity and nano Ag decoration further enhanced the photoactivity *via* the surface plasmon resonance. The function of nano Ag decoration was attributed to: 1) as electron sink reducing the recombination of electrons and holes in the surface of ZnO and 2) as a photo-sensitizer extending the utilization of the visible light. A high quantum yield of 8% was observed at wavelengths <400 nm and only 0.1% at wavelengths ∼470 nm. In the absence of oxygen, ethane was produced owing to the oxidative dehydrogenation of methane and, if ethane further abstracts hydrogen, the generation of ethylene may occur. In the flow mode under oxygen-free conditions a methane conversion of 0.35% and a selectivity of 89.47% for ethane was observed (Chen et al., 2016).

The direct combination of hydrogen evolution from water and methane conversion in a photocatalytic system was performed at 25°C using Pt nanoparticles supported on TiO_2_ as photocatalysts, which were prepared by irradiation of UV light (254 nm) with different mass fraction of Pt (from 0.1 to 2%) (Table 2, entry 7). The activity of Pt/TiO_2_ photocatalysts increased with the increase of Pt content until 1.5% being H_2_, C_2_H_6_ and CO_2_ the main products formed. For all cases, minor quantities of C_2_H_4_ and CO were also observed. The authors considered that the synergistic effect between the two reactions brings up the considerable quantum efficiencies being 4.7% for H_2_ production and 3.3% for CH_4_ conversion. Thus, the synergistic effect of the addition of Pt on TiO_2_ was an efficient way to improve the yields of both H_2_ and C_2_H_6_ products due to the adequate utilization of photo-induced electron and holes, which contributed to the generation of H_2_ and the conversion of CH_4_, respectively (Yu et al., 2017).

The direct combination of hydrogen evolution from water and methane conversion in a photocatalytic system was also performed using Pd/TiO_2_ with different Pd content (0.1–5 wt%) as photocatalysts (Table 2, entry 8). It was shown again that the efficient conversion of methane under ambient conditions and the photocatalytic splitting of water into H_2_ could be introduced into one photocatalytic system achieving the simultaneous utilization of photo-induced electron and hole. Using only TiO_2_ as photocatalyst the CH_4_ conversion was very low and CO_2_ was formed preferentially. The introduction of 0.1% of Pd improved H_2_ production and the CH_4_ conversion and C_2_H_6_ was preferentially formed. Increasing the Pd content (0.5, 1 and 1.5%) the H_2_ production and the CH_4_ conversion also increased, together with increasing of C_2_H_6_ and a little increasing of CO_2_. More additions of Pd (2, 3 and 5%) practically do not increase the H_2_ production and CH_4_ conversion. Thus, the addition of Pd changed the selectivity of the production of C_2_H_6_ and CO_2_, but amounts above 1.5% of Pd barely vary the selectivity (the highest was 72.6%). Considering the desired product (C_2_H_6_) 1.5%-Pd/TiO_2_ was considered as the best photocatalyst. It was determined that the photo-induced electron was used mostly for the production of H_2_, while the hole was used for the conversion of CH_4_. In this manner, the photoinduced electrons and holes can be effectively separated and adequately utilized providing a new strategy for photocatalytic reaction, and brought up considerable quantum efficiencies, which is still low (ca. 5%). The Pd/TiO_2_ catalyst showed excellent property in this novel synergistic photocatalytic pathway between H_2_ production from water and coupling of CH_4_ to ethane. The •CH_3_ radical was demonstrated to play an important role the forming of C_2_H_6_, being the highest selectivity reaching 72.6%. The authors considered that the work provided a new strategy for photocatalytic reaction improving the quantum efficiencies for electron of 2.83% and for hole of 2.76% (Yu and Li, 2017).

The photocatalytic conversion of methane was studied on titanate nanotubes (TNT) and TiO_2_ anatase modified with Au and Rh nanoparticles (Table 2, entry 9). Rh/TNT (average Rh nanoparticles 2.8 nm, band gap 3.08 eV) and Au/TiO_2_ (average Au nanoparticles 7.4 nm, band gap 3.04 eV) were prepared by incipient wetness impregnation and the metals were reduced using H_2_. Au/TNT (average Au nanoparticles 3.1 nm, band gap 3.03 eV) was prepared using NaBH_4_ solution as reducing agent. The photocatalysts were tested using a CH_4_–H_2_O–Ar mixture (CH_4_:H_2_O = 1:1 M ratio). The products formed were H_2_, C_2_H_6_, CO_2_, CO and CH_3_OH. In the photocatalytic tests experiments it was observed that the pure TNT support showed low activity in CH_4_ conversion and the addition of metal significantly increased the rate of CH_4_ consumption. The order of methane conversion was Rh/TNT > Au/TiO_2_ > AuTNT ∼ TiO_2_. The formation of hydrogen followed the same tendency with Rh/TNT producing 296 µmol g_cat_^−1^ h^−1^. The major quantities of ethane production (5.8 µmol g_cat_^−1^ h^−1^) were obtained using TiO_2_ and Au/TiO_2_ photocatalysts showing that the deposition of Au on TiO_2_ did not increase the rate of ethane formation. However, the quantity of H_2_ produced on Au/TiO_2_ (191 µmol g_cat_^−1^ h^−1^) was greater than the TiO_2_ (3.8 µmol g_cat_^−1^ h^−1^) support (László et al., 2018).

Au/TiO_2_, PdO_x_/TiO_2_, PtO/TiO_2_, Cu_2_O/TiO_2_ and FeO_x_/TiO_2_ prepared by impregnation and calcination at 400°C were evaluated for the conversion of methane to methanol at room temperature under moderate light irradiation (close to one Sum) in the presence of H_2_O_2_ (Table 2, entry 10). The samples prepared with noble metals (Au, Pd and Pt) showed lower CH_4_ conversions compared to bare TiO_2_ support being the main product CO_2_. The photocatalyst FeO_x_/TiO_2_ exhibited an increase of 1.5 times for CH_4_ conversion enhancing methanol production by around four times when compared to pure TiO_2_. The methane conversion achieved 14.9% after 3 h of reaction. The yield was 1,056 µmol g_cat_^−1^, that correspond to 18 mol of methanol per mole of Fe. The superior activity of the FeOx/TiO_2_ photocatalyst at ambient conditions was attributed to the efficient electron transfer from TiO_2_ to iron species, a lower overpotential for H_2_O_2_ reduction on iron species and suppression of the oxygen reduction reaction that would change the selectivity to CO_2_, decreasing methanol formation (Xie et al., 2018).

Pt, Pd, Au and Ag (0.1 wt%) loaded on ZnO and TiO_2_ were prepared by borohydride reduction method and tested in the photocatalytic methane oxidation reaction at room temperature in the presence of water and under 2 MPa CH_4_ and 0.1 MPa O_2_ (Table 2, entry 11). Among all photocatalysts Au/ZnO showed superior performance leading to the formation of methyl hydroperoxide, methanol and formaldehyde as the main products with minor quantities of CO_2_ when compared to others photocatalyts. The efficient oxidation of methane was attributed to the powerful ability of noble metal/ZnO photocatalysts to activate simultaneously CH_4_ and O_2_ molecules in aqueous solution generating •CH_3_ and •OOH radicals through of holes and electrons, which lead to the formation of oxygenates liquid products. Conducting experiments using isotopically labeled oxygen and water revealed that molecular O_2_ was the source of oxygen for direct CH_4_ oxidation (Song et al., 2019a).

The photocatalytic oxidative coupling of methane under mild conditions was performed using CO_2_ as oxidant and Ag/TiO_2_ (0.3, 0.5, 1.0 and 1.5% Ag) as photocatalysts prepared by borohydride reduction (Table 2, entry 12). The reaction was carried out in a microautoclave under 2 MPa of pressure in the presence of sunlight. Blank experiment without adding catalyst or in the absence of light showed no products. Using bare TiO_2_ as photocatalyst only a small quantity of CO was detected while the addition of Ag (1 wt%) resulted in the production of 1.149 µmol g_cat_^−1^ h^−1^ of CO and 686 µmol g_cat_^−1^ h^−1^ of C_2_H_4_. In order to understanding the photocatalytic process further experiments were performed and it was concluded that the performance was a result of the synergy of visible-light inducing Ag LSPR effect and UV-light induced TiO_2_ photoexcitation effect, as well as the preferential adsorption of CO_2_ on TiO_2_ and CH_4_ on Ag (Li et al., 2019).

The direct selective photocatalytic conversion of methane into carbon monoxide with only small quantities of CO_2_ was performed in a batch reactor with a quartz window under 0.3 MPa of CH_4_ and 0.1 MPa of air at room temperature (Table 2, entry 13). The photocatalysts used were noble or transition metals (M = Ag, Pd, V, Fe, Ga, Ce, Co, Cu and Zn) species highly dispersed on tungstophosphoric acid, which was dispersed as a thin layer of 1–2 nm over titanium oxide (M-HPW/TiO_2_). In the absence of light no products were observed for all photocatalysts. Using HPW, TiO_2_ and HPW/TiO_2_ as photocatalysts the production of CO and CO_2_ were very low, being CO_2_ preferentially formed. Among the M-HPW/TiO_2_ photocatalysts the material containing Zn showed the best performance producing 429 μmol g^−1^ h^−1^ of CO and 85 μmol g^−1^ h^−1^ of CO_2_ (CO selectivity of 84%). *In-situ* FTIR and XPS suggested that the catalytic performance could be attributed to zinc species highly dispersed on HPW/TiO_2_, which undergo reduction and oxidation cycles during the reaction and the reaction proceeds *via* intermediate formation of methyl carbonates (Yu et al., 2019).

The direct functionalization of methane into ethanol *via* photocatalysis was reported over Cu modified polymeric carbon nitride (Cu-PCN) (Table 2, entry 14). The Cu-PCN photocatalysts were prepared by dissolution of urea and copper chloride in water and after this, the solution was evaporated resulting in a slurry that was treated at 550°C in air. Blanks experiments revealed that no products were formed without photocatalyts or without methane. Using 20 mg Cu-PCN as photocatalysts in the presence of CH_4_ and H_2_O the following quantities of products were observed after 1 h: C_2_H_5_OH = 21 μmol g^−1^ h^−1^, CH_3_OH = 5.5 µmol g^−1^ h^−1^, C_2_H_6_ = 13.9 μmol g^−1^ h^−1^, H_2_ = 7.0 μmol g^−1^ h^−1^ and CO = 2.7 μmol g^−1^ h^−1^. The authors considered that despite the conduction band of Cu/PCN was suitable for H_2_ evolution, no H_2_ was detected in the methane-free experiment thus excluding H_2_O splitting. They consider that Cu-PCN could manage the generation of H_2_O_2_ and its decomposition to produce •OH radicals, of which Cu species were also active sites for methane adsorption and activation. This avoided excess •OH radicals for over-oxidation and facilitated methane conversion (Zhou et al., 2019).

A continuous photocatalytic oxidative coupling of methane at room temperature and atmospheric pressure was described in a flow system reactor (Table 2, entry 15). The Pt and CuO_x_-decorated TiO_2_ photocatalyst denoted as CuPt/TiO_2_ was prepared first introducing Pt by photodeposition and then Cu was incorporated by wet impregnation method. The photocatalysts were prepared with different quantities of Pt and Cu. The optimized Cu_0.1_Pt_0.5_/TiO_2_ photocatalyst showed the highest yield (6.8 μmol h^−1^) of C_2_ products (ethane and ethene, ethane selectivity >90%), which was greater than the obtained with Pt/TiO_2_ (1.07 μmol h^−1^) and Cu/TiO_2_ (1.9 μmol h^−1^). On the other hand, CO_2_ was also formed on these reactions and about 7.5 μmol h^−1^ was obtained using bare TiO_2_, while in the presence of Cu_0.1_Pt_0.5_/TiO_2_ its quantity increased only 20% (∼9 μmol h^−1^). The authors proposed that upon light irradiation electrons were excited from VB of TiO_2_ to its CB and then migrate to Pt, while holes could be transferred to the VB of CuO_x_ retarding their recombination and lowering the oxidation potential of photoinduced holes to avoid deep dehydrogenation and over-oxidation. Thus, C–H bond of methane molecules were broken by the holes in the VB of CuO_x_ species to form methyl radicals that combining forming ethane molecules. The O_2_ were reduced by electrons from Pt sites to form the superoxide anion radical (O_2_•^−^) that react with protons forming H_2_O (Li et al., 2020).

The non-oxidative coupling of methane was studied using M/TiO_2_ (M = Au, Ru, Rh, Pd, Ag, Ir and Pt) as photocatalysts, which were prepared through a photodeposition method (Table 2, entry 16). The reactions were performed in a continuous-flow photocatalytic system. No hydrocarbon products were detected for the NOCM process without light irradiation or replacing CH_4_ by argon even under light irradiation. In the presence of TiO_2_ the methane conversion was very low. In contrast, all M/TiO_2_ photocatalysts were active under light irradiation showing the following order of activity: Au > Ag > Ru > Pd > Ir > Pt > Rh producing 16.3 > 5.2 > 4.3 > 4.2 > 1.9 > 0.8 mmol_C2H6_ g_metal_^−1^ h^−1^, respectively. Among all photocatalysts, Au/TiO_2_ showed the best performance yielding 81.7 µmol g_cat_^−1^ h^−1^ without the addition of H_2_O or any other oxidants. C_3_H_8_ was also produced over Au/TiO_2_, but the yield was very small. The equimolar amount of H_2_:ethane of 1:1 was produced over Au/TiO_2_. The best performance was attributed to the easy transport of photoelectrons from TiO_2_ to Au inhibiting the photoelectron-hole recombination. *In situ* FTIR measurements and DFT calculations revealed that CH_4_ was transformed into methyl radicals to form ethane *via* a methyl anion activation process (Lang et al., 2020).

The methane dry reforming (CH_4_ + CO2 → 2CO + 2H_2_) at room temperature was performed using as photocatalysts Cu-Ru/MgO-Al_2_O_3_ prepared with different Cu-Ru loadings by co-precipitation (Table 2, entry 17). For pure Cu nanoparticles the initial reaction rate was approximately 50 μmol CH_4_ g^−1^ s^−1^, however when an extremely low fraction of Ru (Cu_19.95_Ru_0.05_) was added, the reaction rate increased 2.5 times (128 µmol CH_4_ g^−1^ s^−1^) and the H_2_/CO selectivity value increased to 0.8. Slightly increasing the Ru loading to 0.1 and 0.2 resulted in an increase of reaction rates (between 200 and 275 µmol CH_4_ g^−1^ s^−1^) and H_2_/CO selectivity values were close to 1. Increasing the Ru loading to 0.5 although the activity increased, the selectivity values decreased. The substantial differences in the photocatalytic activity with different Ru quantities (from 0.05 to 0.50) was attributed to monodispersed Ru atoms (single-atom) on the Cu nanoparticle. It was proposed light-excited hot carriers together with single-atom active sites cause the observed performance, that is, the small plasmonic Cu provide strong light absorption generating hot carriers under illumination, while single-atom Ru sites on the Cu surface offer high catalytic activity (Zhou et al., 2020).

Recently, a photocatalytic conversion of methane to ethylene under mild conditions was reported using AuPd/ZnO nanoporous as photocatalysts prepared by depositing pre-synthesized AuPd core-shell nanorods on the porous of ZnO (Table 2, entry 18). Au nanorods were chosen as the plasmonic metal nanostructure and Pd was selected as a modifier to modulate the dehydrogenation capability to improve C_2_H_4_ selectivity. Using pure ZnO as photocatalyst practically no products were formed. Au/ZnO and Pd/ZnO showed low activity and C_2_H_4_ selectivity; however, introducing Pd into the lattice of Au the photocatalytic performance and especially C_2_H_4_ selectivity increased strongly. As a result, a methane conversion of 536 μmol g^−1^ and a C_2_^+^ compounds selectivity of 96% (39.7% C_2_H_4_ and 54.9% C_2_H_6_ in total produced C_2_^+^ compounds) was obtained using the optimized AuPd_2.7%_/ZnO photocatalyst after 8 h of irradiaton. Based on *in situ* characterizations, it was revealed that methane molecules were first dissociated into methoxy on the surface of ZnO under the assistance of Pd and the methoxy intermediates were further dehydrogenated and coupled with methyl radical into ethoxy, which were subsequently converted into ethylene through dehydrogenation (Jiang et al., 2021).

Analyzing the data in Tables 1, 2 it could be seen that the products formed in the photocatalytic conversion of methane are greatly influenced by the conditions under which the reactions are carried out, such as:

1) *In the presence of O*_*2*_ *or the use of oxidizing agent like H*_*2*_ *O*_*2*_: the oxygenated products like CH_3_OH, HCOOH, CH_3_COH, CH_3_OOH, HCHO, CO and CO_2_ were formed (Table 1, entries 4 and 6 and Table 2, entries 10, 11 and 13).
2) *In the absence of water and oxygen*: the non-oxidative coupling of methane reaction was favored forming C_2_H_6_ and C_2_H_4_ (Table 1, entries 1 and 2 and Table 2, entries 3, 6, 16 and 18).
3) *In the presence of water and absence of oxygen*: a greater diversity of products such as CH_3_OH, CH_3_CHO, CH_3_CH_2_OH, C_2_H_6_, CO, CO_2_ and H_2_ could be formed (Table 1, entries 3 and 5 and Table 2, entries 1, 2, 4, 5, 7, 8, 9 and 14). The advantage of carrying out the reaction in the presence of water is that the evolution of hydrogen can be combined with the conversion of methane (Table 2, entries 4, 7, 8, 9).
4) *In the presence of CO*_*2*_: the formation of C_2_H_4_ and CO through the oxidative coupling of methane was described, where CO_2_ works as an oxidizing agent (Table 2, entry 12) and the formation of syn gas CO: H_2_ (1:1) by dry reforming of methane (Table 2, entry 17).

Another important point is that the use of different photocatalysts affect the methane conversion and the selectivity of the products formed when the reaction are carried out under similar conditions. For example, see Table 2, entries 7, 8 and 9, where the reactions were performed in the presence of water and absence of oxygen using Pt/TiO_2_, Pd/TiO_2_ and Rh/TNT photocatalysts, respectively. In these reactions, the products formed for all photocatalysts were C_2_H_6_, CO_2_ and H_2_, however, the methane conversions and products selectivity were different. Another example is observed for reactions performed in the absence of water and oxygen (Table 2, entries 16 and 18) where the Au/TiO_2_ and Pd-Au/TiO_2_ photocatalysts were more selective for the production of C_2_H_6_ or C_2_H_4_. Also, some reactions were favored using different photocatalysts, for instance, the reactions carried out in the presence of CO_2_ using the same CH_4_:CO_2_ molar ratio of 1:1 (Table 2, entries 12 and 17). The oxidative coupling of methane was favored using Ag/TiO_2_ as photocatalyst (Table 2, entry 12) and dry reforming of methane using Cu-Ru/MgO-Al_2_O_3_ photocatalyst (Table 2, entry 17). On the other hand, it should be noted that the activities of the photocatalysts for methane conversion are still quite low (µmol g^−1^ h^−1^). Thus, due to the different conditions used in these reactions (type of reactor, light source, etc.) and techniques for detection and quantification of the products formed, a quantitative comparison of these data may not be very accurate.

## Conclusions and Perspectives

Studies on the photocatalytic conversion of methane at low temperatures have attracted the attention of the scientific community in recent years with the objective of the production of more valuable products (ethane, ethylene, methanol, CO, hydrogen) reducing the energy consumption and the environmental impacts. The photocatalysts that have been most used in these reactions are semiconductors like TiO_2_, ZnO, Ga_2_O_3_ and WO_3_ and hybrid metal-semiconductor materials, where metals like Pt, Pd, Au, Ag, Cu and Fe have been the most used. The most studied reactions are the oxidative and non-oxidative coupling of methane, the direct conversion of methane to methanol and steam and dry reforming of methane. It is important to point out the difficulty in establishing proper comparisons among the various works since the operational conditions used are very different (light source, pressure, reactor-type, etc.). In addition, studies of stability and deactivation of photocatalysts are also absent in most works. The combination of metal and semiconductor has proved to be a good strategy to develop more active photocatalysts. This is basically due to the improvement of charge separation (photoelectrons and holes) pulping the electrons to surface for reaction, the activation of methane by metals and the plasmon resonance effect of some metals. Therefore, the development of more active photocatalysts should take into account the increase in light absorption (mainly in the visible region), the separation of charges and the surface reactions. This involves some strategies and methods to control the composition, sizes and morphologies of the metals and the semiconductors, the doping of the semiconductors and the interactions between them, for instance, depositing metals on specific facets of the semiconductor, joining semiconductors to facilitate the separation of photogenerated charge carriers, etc. The metal organic framework (MOF) has the property of adsorbing large amounts of methane and, therefore, can be an interesting for developing hybrid photocatalysts for methane conversion (Shah et al., 2020). However, although the knowledge in this area has advanced, the possibilities of combinations are endless and there is a plenty of room to be investigated and improved.
